# Study on the Bonding Performance of BFRP Bars with Seawater Sand Concrete

**DOI:** 10.3390/ma17030543

**Published:** 2024-01-23

**Authors:** Guohao Guan, Xuezhi Wang, Ming Xin, Chuanwu Sun, Qingqing Zhang, Jingjing He

**Affiliations:** 1 School of Civil and Architectural Engineering, Liaoning University of Technology, Jinzhou 121001, China; 15090254086@163.com (G.G.); xmmyemail@163.com (M.X.); scw18914870102@163.com (C.S.); 13633751561@163.com (Q.Z.); 2 PowerChina Northwest Engineering Corporation Limited, Xi’an 710100, China; hejing_86@126.com

**Keywords:** BFRP bars, seawater sand concrete (SSC), bonding performance, bond–slip curve, constitutive relationship model

## Abstract

A total of 66 sets of pullout specimens were prepared to investigate the bonding properties of basalt fiber-reinforced polymer reinforcement (hereinafter referred to as BFRP) with seawater sand concrete (hereinafter referred to as SSC). The volume dosages of mono-doped glass fibers and mono-doped polypropylene fibers were 0.1%, 0.2%, and 0.3%; the total volume dosage was set to be constant at 0.3%; and the doping ratios of the hybrid fibers were 1:2, 1:1, and 2:1. The effect on the bonding performance of BFRP reinforcement with SSC was studied on the condition of the diameter D of the BFRP reinforcement being 12 mm; the bond length of SSC being 3D, 5D, and 7D; and the surface characteristics of the reinforcement being sandblasted and threaded. The research showed that due to internal cracks in the matrix, salt crystals in the pores, chloride salts with high brittleness and expansion, as well as sulfate corrosion products such as “Frederick salts” in SSC, the concrete became brittle, resulting in more brittle splitting failures during the pullout test. Doped fibers can increase the ductility effect of concrete, but the bonding effect between the threaded fiber reinforcement and the SSC was not as good as that of the sandblasting group. When the bond length was 5D, the bonding effect between the BFRP reinforcement and SSC was the best, and the bonding performance of the experimental group with doped fibers was better than that of the threaded group. Finally, by combining the ascending segment of the Malvar model with the descending segment of the improved BPE model, a constitutive relationship model suitable for the bond–slip curve between BFRP reinforcement and SSC was fitted, which laid a theoretical foundation for future research on SSC.

## 1. Introduction

The civil construction industry is booming with the expansion of the global economy since the early 21st century. However, environmental problems and the unsustainable development of resources brought about by the rapid development of the industrial era have become increasingly significant [1,2,3]. In the future, the development mode of using natural river sand and fresh water as the main raw materials for construction will be changed, so it has become particularly important to find construction resources that can replace these materials [4]. The development of coastal cities and marine engineering construction relies on the deployment of resources from the mainland. If the transportation distance, time, safety, and other issues are taken into account, it is inevitable that the concept of locally sourced materials will be widely promoted [5]. The Earth is rich in seawater resources and huge amounts of sea sand. Effectively utilizing seawater sand in the construction industry will lead to a profound change and promote a virtuous cycle of good development in marine engineering construction and coastal city construction, which has important and profound practical significance for building an environmentally friendly society and deepening the concept of sustainable development [6,7].

Many scholars have conducted relevant research on the basic mechanical properties of SSC, which shows that SSC has excellent basic mechanical properties and a higher early strength than that of normal-strength concrete. Its long-term strength develops slowly but generally meets the strength requirements of concrete [8,9,10,11,12,13]. Currently, there is not much research on the bonding performance between SSC and reinforcement materials. Some studies have pointed out that although the chloride and sulfate components contained in SSC can play a certain role as early strength agents in concrete, the erosion deterioration effect for steel reinforcement is quite obvious. Reducing the bonding properties between concrete and reinforcement will have a serious impact on the mechanical properties and durability of concrete components and even buildings [14,15,16]. Therefore, seeking a material with excellent mechanical properties of steel reinforcement and certain corrosion resistance has become the research focus of many scholars. The use of fiber-reinforced polymers (FRPs) has become a hot topic for scholars because of their light weight, high strength, and good corrosion resistance [17,18,19,20]. The bonding performance is the basis of ensuring that the FRP reinforcement and SSC have good synergistic working conditions [21,22]. In 2011, Prof. Teng Jinguang’s research team proposed, for the first time in Hangzhou, developing combined FRP reinforcement and SSC structure technology for offshore engineering and island construction [23], which was internationally promoted at ACMSM23 (23rd Australian Conference on Mechanics of Structures and Materials) and other conferences.

Zeng et al. [24] studied the variation rule of the bonding performance between GFRP reinforcements with diameters of 6 mm, 10 mm, and 16 mm and SSC at bond lengths of 2.5 d and 5 d. The study showed that too large or too small reinforcement diameters and bond lengths would reduce the bond strength. Doping with 0.5% PE fiber can improve the bond strength by 3%~10%, but more than 0.5% PE fibers will affect the development of bond strength; in addition, the bond strength of the combined GFRP reinforcement and SSC structure is similar to that of normal-strength concrete. This conclusion is consistent with the research results of Liao et al. [25]. Yu et al. [26] studied the bond degradation pattern of AFRP reinforcement and concrete specimens after freshwater immersion and chloride and sulfate erosion. They showed that the bond strength degradation exhibited after 300 days of freshwater immersion was the most obvious, and it was higher than that of chloride and sulfate erosion of the reinforcement. It can be concluded that the AFRP reinforcement, which replaces the steel reinforcement and is placed inside the SSC, can better fulfill the stressing task of concrete members. Wang et al. [27] pointed out that in dry environments, CFRP reinforcement and SSC components mainly experience interlayer concrete failure during pullout tests, specifically in splitting failure; meanwhile, the mode of bond failure after immersion in seawater is mainly the pullout failure of the reinforcement. The reason for this is that after seawater immersion and erosion, the surface of the reinforcement material at the interface between the CFRP reinforcement and concrete components becomes damaged, and cracks appear in the concrete aggregate mortar due to erosion. This makes the bond strength between CFRP reinforcement and concrete being lower than the strength of the concrete, and ultimately, the pullout failure of the reinforcement occurs. Wu et al. [28] concluded from a comparative study by preparing BFRP bars, SSC and normal-strength concrete pullout specimens that the bond strength between BFRP bars and SSC was always higher than that of normal-strength concrete specimens. This is because the SSC matrix provided a chloride environment for the reinforcement, which led to a chemical reaction between the reinforcement and the interfacial concrete, and it increased its bonding performance; on the other hand, a series of geometrical expansions of the reinforcement occurred after erosion, which increased the interaction between the reinforcement and the interfacial concrete, thus increasing the bond strength. Zhang et al. [29] crushed the coral reefs and screened the suitable particle size to be used to replace the natural aggregates to save resources. The change rule in the bond strength of BFRP bars and seawater coral aggregate concrete specimens was characterized by studying the structural durability of the two in the marine environment. The study showed that the bond strength of the specimen was increased under the conditions of dry–wet cycling and immersion in seawater. The reason is that when the specimen was exposed to seawater, the hygroscopic expansion of BFRP bars increased the mechanical anchoring effect between the reinforcement and the concrete, thereby improving the bond strength between the two. After the specimens were subjected to dry–wet cyclic failure in seawater at 45 °C and 60 °C, the bond strength between BFRP bars and seawater coral concrete decreased by approximately 6.5% and 12.0%, respectively. Li et al. [30] studied the static and dynamic tensile of BFRP bars after embedding the BFRP bars inside the SSC concrete and completing the exposure test in seawater. The study showed that the tensile properties of BFRP bars were negatively correlated with the strain rate, but the strain effect of the specimen at the time of failure was small. This provides a theoretical basis for future ocean engineering and the construction of coastal cities.

In conclusion, the excellent performance of FRP reinforcement in SSC provides a strong basis for replacing the steel reinforcement as the main tensile reinforcement material in the construction of marine engineering. However, in the actual engineering practice, not only the quality and effect of engineering construction but also its construction cost is an important factor that cannot be ignored. This paper takes into account that although AFRP reinforcement, CFRP reinforcement, and GFRP reinforcement show good bonding performance and corrosion resistance in SSC, the bonding performance between the BFRP bars and the SSC is worse than those of the above three materials, and the corrosion resistance of BFRP is even better than that of GFRP in seawater immersion erosion. Therefore, in this paper, BFRP bars are selected as the main reinforcement of this test, and the pullout specimen prepared by adding a certain amount of glass fiber (GF) and polypropylene fiber (PPF) is deeply studied. The development law of bonding property is different from that of the specimen without fiber. Therefore, the biggest innovation of this paper is the use of seawater sand instead of the traditional freshwater river sand preparation of concrete, in line with the sustainable development of the environment, and the study of BFRP tendons and seawater sand concrete in a variety of factors under the influence of the change rule of the bonding properties of the concrete for the development of the construction of offshore industry to provide certain theoretical guidance.

## 2. Test Overview

### 2.1. Test Materials

**Figure 1:**
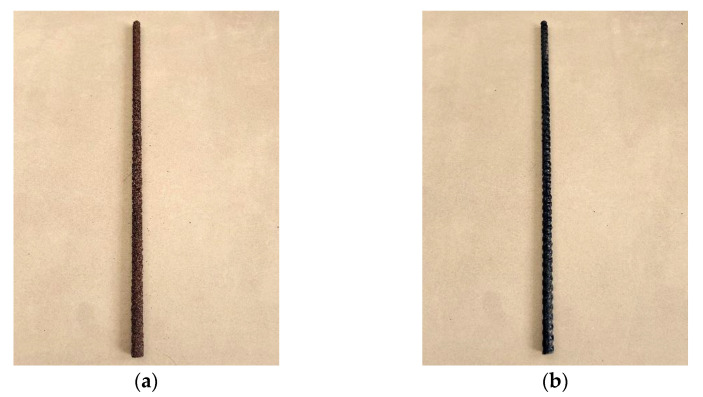
Morphology and appearance of BFRP bars. (**a**) Surface sandblasting; (**b**) Thread ribbed.

**Figure 2:**
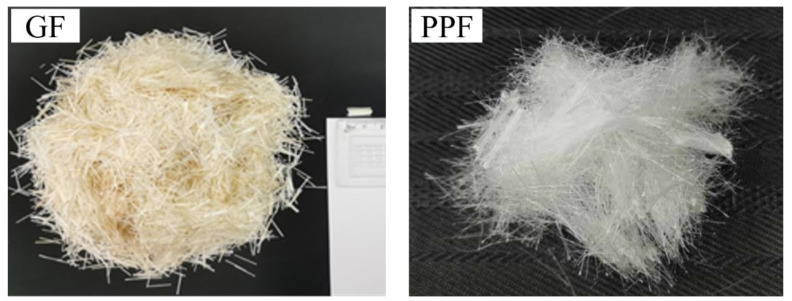
Fiber appearance characteristics.

(1) The extruded ribbed and sandblasted BFRP bars produced by Jiangsu Nanjing GMV New Materials Technology Development Co., Ltd. (Nanjing, China) are selected. The performance indexes and appearance characteristics are shown in Table 1 and Figure 1, respectively.
(2) Seawater: Seawater from the waters near Bijiashan, Jinzhou, Liaoning is selected. The specific ion components in seawater are shown in Table 2.
(3) Sea sand: The fine aggregate sea sand is obtained by soaking the river sand in undisturbed seawater. The river sand is medium sand with a fineness modulus of 2.36 and a mud content of 2.6% produced by a local company in Jinzhou. The river sand was poured into a steel tank, and the undisturbed seawater was added to cover the upper surface of the river sand by 20 mm. To ensure the seawater is fully immersed in various ion components, it is stipulated that the seawater shall be changed every three days and the sand shall be soaked continuously for 30 days.
(4) Cement: The cementing material selects PO42.5 cement of Bohai brand produced in Huludao, Liaoning Province. The specific chemical composition and performance are shown in Table 3 and Table 4.
(5) Coarse aggregate: An aggregate of natural sand and gravel is selected. The basic physical and mechanical properties are shown in Table 5.
(6) Fiber: GF and PPF adopt the fibers produced by Huierjie New Material Technology Co., Ltd. (Xiangyang, China) and Ningxiang Building Materials Co., Ltd. (Changsha, China) respectively. The physical properties and appearance characteristics are shown in Table 6 and Figure 2, where ZrO_2_ in GF accounts for 16.7%, which is larger than the 14.5% generally found in fiber products in the industry. It enhances the alkali resistance of the fiber and reduces the probability of fiber erosion by alkaline ions in seawater sand.

### 2.2. Test Parameters

The test concrete had a strength grade of C40. A total of 66 pullout specimens were set up for the bonding performance test between BFRP bars and SSC, 3 in each group. Both GF and PPF fibers took the volume dosages of 0.1%, 0.2%, and 0.3% as the main variable parameters. The hybrid fibers composed of GF and PPF fibers took a total volume fraction of 0.3% as well as 1:2, 1:1, and 2:1 blending ratios of GF to PPF fibers as the main variation parameters.

### 2.3. Preparation of Specimens

**Figure 3:**
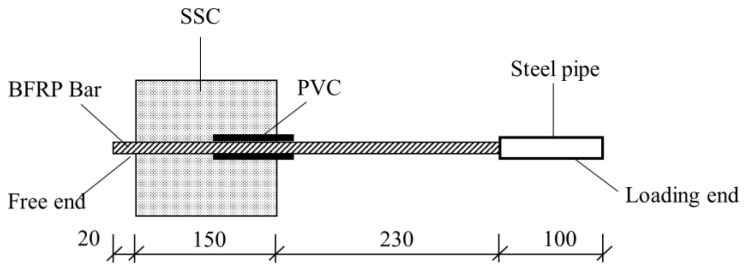
Diagram of pullout test specimen.

The central pullout test of BFRP bars and SSC was mainly designed following the “Standard for Test Methods of Concrete Structures” (GB/T50152-2012) [31]. The test in this chapter selected a 150 × 150 × 150 mm central pullout specimen, and the length of the fiber reinforcement was set to 500 mm. Upon determining the specific bonding zone length of each specimen, an additional 20 mm length was reserved at the free end for later collection of free end slippage data. A PVC pipe was put on as a non-bonded area, and the epoxy resin gel was poured on both ends of the PVC pipe to fix it tightly to prevent the PVC pipe from falling off during the preparation of the specimen or the error in the length of the bonded area as well as to prevent the reinforcement in the later test process from squeezing back and forth the pipe wall, which will cause the stress concentration, leading to greater errors in the test. Due to the smooth surface of the PVC pipe, the friction between the PVC pipe and the concrete can be disregarded during the actual test process. It was approximately considered that the ultimate stress on the specimen was the bonding stress between the BFRP bars and the SSC. After curing for 28 days under standard curing conditions, the pull out specimen was taken out, and a 100 mm steel pipe was put on the loading end and sealed tightly with expansion cement, being cured for more than seven days to prevent the steel pipe from falling off during the test and prevent the fixture from squeezing the reinforcement and causing uneven stress on the surface and inside of the reinforcement, resulting in shear fracture damage. The specific specimen diagram is shown in Figure 3.

### 2.4. Test Equipment and Loading Methods

**Figure 4:**
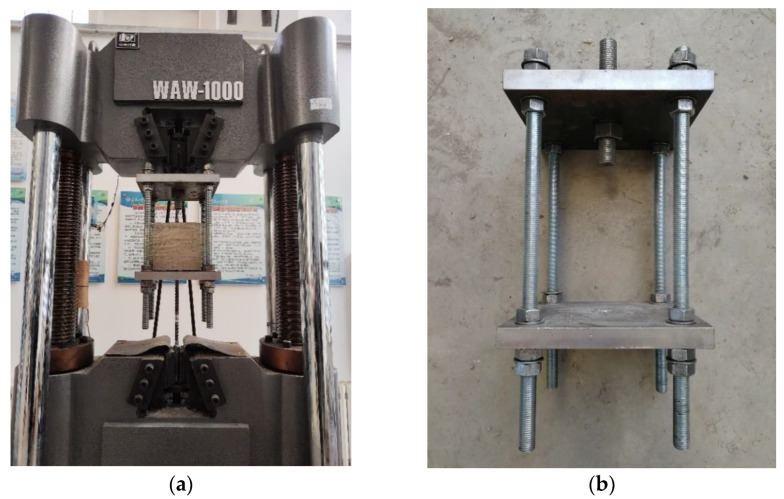
Pull-out test machine. (**a**) Servo universal testing machine; (**b**) counter-force steel plate cage.

The electro-hydraulic servo universal testing machine model WDW-1000 produced by Changchun Xinke and the counter-force steel plate cage for loading the specimen were used, as shown in Figure 4. The test method is as follows: first, the central pull-out specimen after curing was slowly loaded into the steel plate cage-type equipment, and four support bolts of the equipment were adjusted to keep the pressure steel plate horizontal to prevent test errors caused by eccentric force. After adjusting the position of the specimen, the tape was wrapped around the counter-force steel plate cage in several turns to prevent the concrete debris from falling and injuring people in the event of splitting failure. Finally, a linear displacer was placed at the free end to record the data on the relative displacement of the fiber reinforcement. The loading method adopted the displacement control method, and the loading rate was controlled to 1 mm/min.

## 3. Test Results

Generally, the bonding stress between BFRP bars and SSC exhibited a linear distribution along the longitudinal axis of the fiber reinforcement, but some stress concentration will inevitably occur, leading to certain deviations during the test. Therefore, the average bond stress can more accurately reflect the development of bonding performance during the test and the ultimate load that the specimen can withstand. Therefore, the calculation process of bond strength in this test will use Equation (1) [32,33,34], and the average bond strength will be calculated as the actual value of the bond strength between BFRP bars and SSC. The detailed test calculation results are presented in Table 7.

(1) $$\tau =\frac{P}{\pi dl}$$

where

*τ*—average bond strength between BFRP bars and SSC (MPa);

*P*—the ultimate load (N) that the concrete can withstand at the time of failure;

*d*—BFRP bar diameter (mm);

*l*—bond length between BFRP bars and SSC (mm).

**Table 7:** Adhesion performance test data.

Group	No.	Surface Treatment Characteristics	Bond Length	*P*/kN	*τ*/MPa	*s*/mm
1	SSC	R	3D	19.95	14.71	1.51
2	SSC	R	5D	34.23	15.14	1.60
3	SSC	R	7D	45.01	14.2	1.54
4	SSC	S	5D	27.90	12.31	4.20
5	SSC-GF_0.1_	R	5D	47.93	21.20	3.51
6	SSC-GF_0.2_	R	5D	42.81	18.94	3.20
7	SSC-GF_0.3_	R	3D	23.39	17.24	7.80
8	SSC-GF_0.3_	R	5D	41.90	18.53	3.06
9	SSC-GF_0.3_	R	7D	52.41	16.56	2.71
10	SSC-GF_0.3_	S	5D	41.22	16.74	2.09
11	SSC-PPF_0.1_	R	5D	41.36	18.29	3.32
12	SSC-PPF_0.2_	R	5D	48.37	21.39	2.32
13	SSC-PPF_0.3_	R	3D	21.54	15.88	3.66
14	SSC-PPF_0.3_	R	5D	37.19	16.45	3.08
15	SSC-PPF_0.3_	R	7D	51.11	16.15	2.71
16	SSC-PPF_0.3_	S	5D	39.08	17.28	9.52
17	SSC-GF_0.1_ PPF_0.2_	R	5D	35.19	15.56	2.33
18	SSC-GF_0.15_ PPF_0.15_	R	3D	22.70	16.74	3.07
19	SSC-GF_0.15_ PPF_0.15_	R	5D	41.91	18.54	2.34
20	SSC-GF_0.15_ PPF_0.15_	R	7D	53.91	17.03	2.53
21	SSC-GF_0.15_ PPF_0.15_	S	5D	52.17	23.08	4.57
22	SSC-GF_0.2_ PPF_0.1_	R	5D	41.02	18.14	1.39

Note: *P* is the ultimate load, *τ* is the average bond strength, and *s* is the bond–slip displacement.

## 4. Test Analysis

### 4.1. Effect of Different Influencing Factors on Bond Strength

#### 4.1.1. Effect of Fibers on Bond Strength

**Figure 5:**
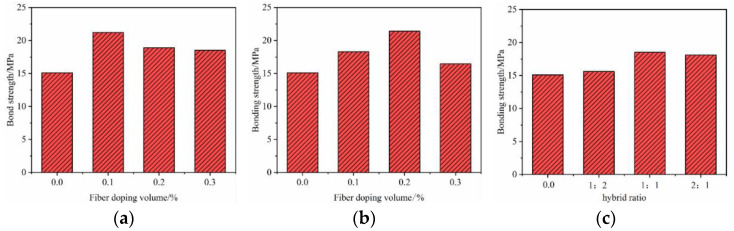
Effect of different fiber dosage on bond strength. (**a**) GF fiber; (**b**) PPF fiber; (**c**) hybrid fiber.

Figure 5 shows the effect of different fiber doping volumes on the bond strength of BFRP bars to SSC concrete. The controlled variable analysis method was used, i.e., the threaded reinforcement with a bond length of 5D was taken as the control variable, and mono-doped GF or 0.1%, 0.2%, or 0.3% of PPF fiber volume doping and 1:2, 1:1, or 2:1 fiber doping ratios were taken as the independent variables. Comprehensively, the bond strengths between BFRP tendons and SSCs showed a continuous trend of increasing and then slowly decreasing with the increase in fiber volume doping and the changes in mixing ratio, and the bond strengths of the fiber-doped specimens were higher than those of the control group with no fiber doped. Taken individually, (1) when the GF content was 0.1%, 0.2%, and 0.3%, the bonding strength increased by 40.40%, 25.17%, and 22.52%, respectively, compared with the control group. It can be concluded from this that for GF with high elastic modulus when the volume content of GF was 0.1%, the effect on improving bond strength was most obvious. Therefore, it can be understood that the improvement of bond strength by GF did not mean that the higher the content, the better. Only an appropriate amount can obtain the ideal improvement effect [35]. (2) When the dosage of PPF was 0.1%, 0.2%, and 0.3%, the bond strength was enhanced by 21.19%, 41.65%, and 8.94, respectively, compared with the control, and it can be concluded that for the low elasticity modulus of PPF, the volume doping of 0.2% PPF has the most significant effect on the enhancement of the bond strength. However, compared with 0.2%, when the dosage is 0.3%, the bonding strength decreased rapidly. It can be concluded that PPF has a better effect on improving the bonding performance of SSC, but it is not suitable for large dosages; (3) As the doping ratio is changed to 1:2, 1:1, and 2:1, the bond strength increases by 3.31%, 22.51%, and 19.87%, respectively. It can be concluded that when the mixing ratio was 1:1, the hybrid fiber had the best effect on improving the bond strength between BFRP bars and SSC, and the GF fiber was better than the PPF in terms of the enhancement of the bonding performance.

**Figure 6:**
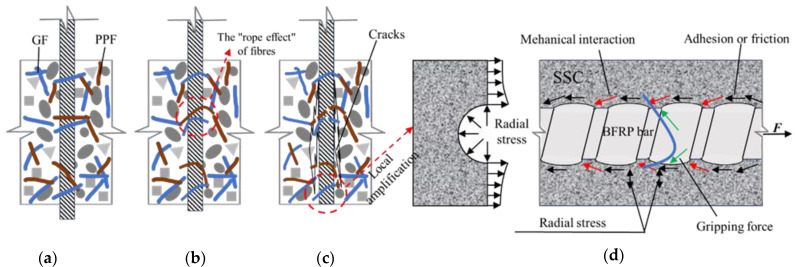
Detailed diagram of the internal fiber reinforcement principle and radial stress distribution of concrete in the pullout specimen. (**a**) Resting state; (**b**) rope effect; (**c**) cracking; (**d**) radial stress distribution diagram.

In summary, whether GF or PPF is individually doped, or both are doped into SSC according to a certain mixing ratio, the bonding performance between BFRP bars and SSC can be enhanced. A certain quantity of GF exhibits the most stable and pronounced effect on improving the bonding performance between BFRP bars and SSC, followed by hybrid fibers, and finally PPF. We found the following reasons: (1) GF has good dispersion and can be evenly and irregularly dispersed in the concrete during the preparation process of the specimen, which can increase the toughness of the specimen [36]. Under the condition of bearing a certain load, the bridging effect between fibers can delay the cracking and failure time of the specimen. In pullout failure, the gripping force between the fibers around the reinforcement and the concrete acts as a “rope” tension (as shown in Figure 6a), which effectively increases the friction between the reinforcement and the concrete, thereby improving the bond strength. (2) The dispersion of PPF is not as good as that of GF, but sufficient stirring can overcome this problem. However, it is precisely because of this deficiency that PPF can establish a more steadfast system within the concrete matrix. The fiber filaments are closely arranged, which can make up for the defects of internal capillary pores and gaps caused by uneven stirring or insufficient vibration in the early stage of the test to a certain extent, thereby densifying the concrete matrix [37] and improving the bonding performance between the reinforcement and the matrix. On the other hand, PPF fibers with low elastic modulus can suppress the occurrence of micro-cracks before they occur in the specimen, delay the cracking time, increase the friction between the reinforcement and the concrete matrix, thereby improving the bonding performance (as shown in Figure 6b). (3) For hybrid fibers, GF with high elastic modulus and PPF with low elastic modulus form a three-dimensional stable system. When loaded, the two fibers coordinate with each other to bear the stress, minimizing the stress concentration. In the early stage of loading, PPF with a low elastic modulus acts as the primary stress-bearing fiber, effectively preventing the initiation and propagation of cracks. When the load continues to increase, it is normal for concrete to crack. At this time, most of the low-elastic modulus PPF on both sides of the crack stops working, and the high-elastic modulus GF bears the main anti-crack task, aiming to delay the rapid and continuous increase and widening of the crack, thereby improving the bonding performance between the reinforcement and the concrete (as shown in Figure 6c). Two fibers cooperate in terms of stress and work complementarily to offset the radial stress generated by part of the reinforcement (as shown in Figure 6d), delay concrete cracking, and also reduce the fatigue that is more likely to occur with one type of fiber to the greatest probability [38]. (4) In the process of densifying the SSC matrix, doping the fibers in three different ways will also hinder the erosion of the fiber reinforcement by harmful ions in seawater and sand to a certain extent, ensuring that the fiber reinforcement is in a normal working state to the greatest extent.

#### 4.1.2. Effect of Bond Length on Bond Strength

**Figure 7:**
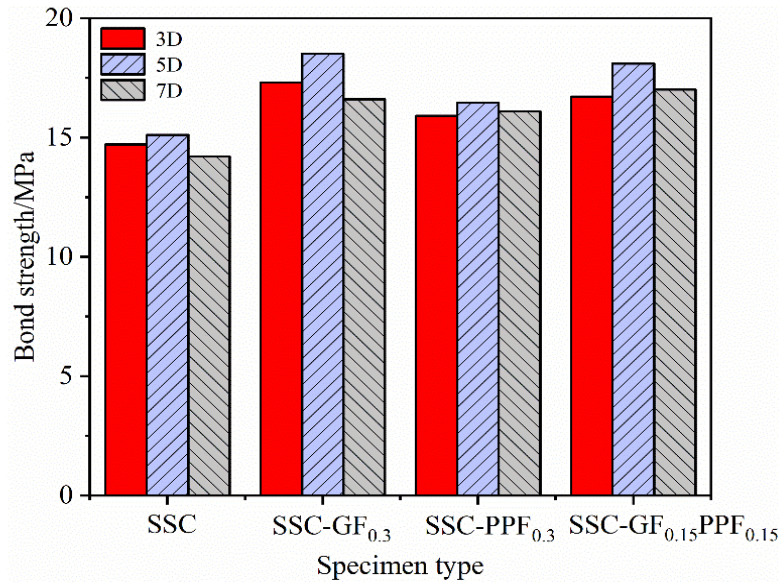
Effect of different bond lengths of BFRP bars on bonding strength.

Figure 7 shows the control variable analysis method, set the fiber content to 0.3%, and adopted the surface treatment feature of BFRP bars with threads to study the changing pattern of bond strength between BFRP bars and SSC center pullout specimens under the effect of different bond lengths. The test showed that regardless of whether fiber was added or not, the development of bond strength generally conformed to the trend of first increasing and then decreasing as the bond length of the reinforcement increased. Excessive bond length led to the loss of bonding strength, which was consistent with the research results of many scholars [39,40,41,42], and the effects of different types of fibers on bond strength were slightly different. Specifically, the 3D specimens with bond length in each group of specimens were taken as the control group, and the bond lengths of the SSC group with no fiber doped were 5D and 7D; the bonding strength of the specimens was improved by 2.72%, and −3.40%; the SSC-GF0.3 group was improved by 6.94% and 4.05%; the SSC-PPF0.3 group was improved by 3.77% and 1.56%; and the SSC-GF0.15PPF0.15 group was improved by 10.78% and 1.86%, respectively. It can be concluded that when the bond length is 5D, the bonding strength improvement effect of BFRP bars and SSC is the most obvious in each doping ratio. This is the optimal bond length for the reinforcement in this test. When the bond length exceeds 5D, the bonding strength shows varying degrees of decline. The reason is that when the reinforcement is subjected to load, the stress is distributed linearly along the length of the reinforcement. When the bond length exceeds the optimal value, due to defects such as uneven mixing and loose vibration in the concrete, as well as defects in the reinforcement, the stress concentration occurs near the bonding interface, causing the bonding stress to transform into nonlinear development and reducing the strength; on the other hand, as the bond length is greater than the optimal bond length and continues to increase, the high-pressure zone between the reinforcement and concrete becomes smaller, so the bond strength decreases.

#### 4.1.3. Effect of Surface Characteristics on Bond Strength

**Figure 8:**
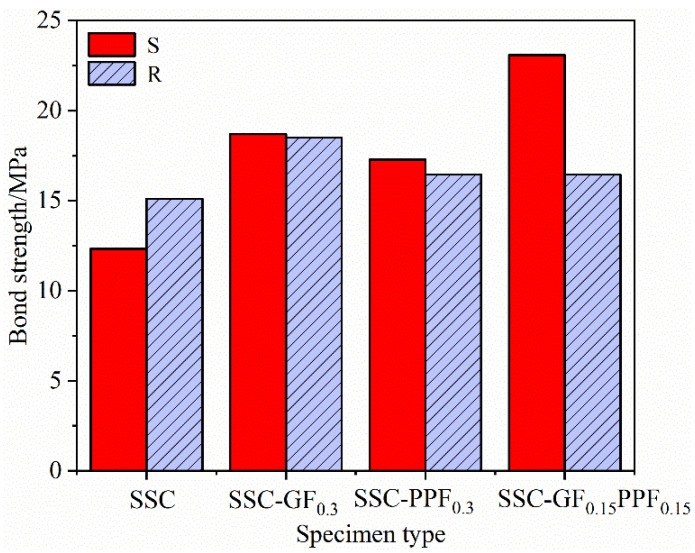
Effect of different surface characteristics of BFRP bars on bond strength.

Figure 8 reflects several groups of mixed proportion specimens. The fiber doping content was set to 0.3% and the bond length was 5D, and the effect of BFRP bars on the bond strength under the effect of two different surface characteristics of threads and sandblasting was studied. As shown in the figure, except for the SSC control group without fiber doped, the remaining three groups showed that the bond strength of sandblasted reinforcement specimens was higher than that of threaded reinforcement specimens, in which the effect of the hybrid fiber group was the most obvious. In terms of bond strength, the sandblasted reinforcement specimens were higher than the threaded specimens by 39.16%; the sandblasted specimens with mono-doped GF fibers were higher than the threaded specimens by 1.08%; the sandblasted specimens with mono-doped PPF fibers were higher than the threaded specimens by 4.85%; and the sandblasted specimens in the SSC control group without fiber doped were higher than the threaded specimens by25.17%. There are several reasons for this analysis: for the reinforcements with sandblasting treatment on the surface, the existence of fine sand particles increases the specific surface area, so more cement hydration products are likely to penetrate between the particles, which strengthens the chemical cementation force; on the other hand, the surface roughness of sandblasted reinforcement is greater than that of the threaded reinforcement, which increases the friction between the reinforcement and the concrete matrix. The coupling effect of these two factors jointly strengthens the bonding performance between reinforcement and concrete.

### 4.2. Effect of Different Influencing Factors on the Bond–Slip Curve

#### 4.2.1. Effect of Fibers on Bond–Slip Curve

**Figure 9:**
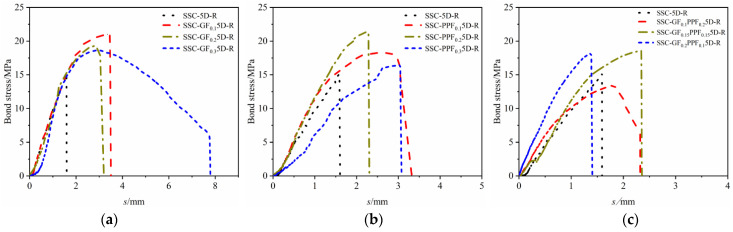
Effect of fiber on bond–slip curve. (**a**) GF; (**b**) PPF; (**c**) hybrid fiber.

Figure 9 shows the bond–slip curve between BFRP bars and SSC when GF is mono-doped. It can be seen from the figure that the bond–slip curve is similar to that of normal-strength concrete. The development rules of the bond–slip curve can be roughly divided into two types. (1) As shown in Figure 9 (a) SSC-GF_0.3_5D-R, (b) SSC-PPF_0.1_-5D-R, and (c) SSC-GF_0.1_PPF_0.2_5D-R, there are two segments of the curve where the pullout failure occurs: the ascending segment and the descending segment. In the ascending segment of the curve, the loading end continues to apply the load. Due to the high elastic modulus of concrete, the bond–slip curve exhibits micro-slip and linear growth, and the bond stress slope is large, which indicates that the stress increases rapidly and the free-end slip displacement is limited. As the load continues to increase, the deformation state of the concrete enters the elastic–plastic stage from the elastic stage, while both the fiber reinforcement and concrete advance into the slip stage. The slope of the bond–slip curve becomes smaller, transitioning from linear growth to nonlinear growth, and it reaches the ultimate stress state. Here, the slope of the bond–slip curve approaches zero. This is because the increasing load causes micro-cracks inside the concrete, especially at the interface between the fiber reinforcement and the concrete matrix. The occurrence and development of micro-cracks gradually weaken the chemical bonding force between the fiber reinforcement and concrete bonding interface [43] and eventually lose it. Here, the displacement speed of the free end is greater than that of the previous micro-slip stage. After entering the descending stage, the chemical bonding force between the fiber reinforcement and concrete fails to work, and the main bonding force is borne by the surface friction of reinforcement and the mechanical snap-in force between the reinforcement and concrete [44]. Here, the free end displacement continues to increase, and the bond–slip curve appears to have a slow downward trend until the fiber reinforcement is pulled out, so the test is over. The reason is that the doped fibers increase the friction between the reinforcement and the concrete matrix. The main principle is that a large number of fibers are distributed in the transition area of the contact surface between the reinforcement and the concrete. The fibers and the reinforcement are entangled with each other and attached to the surface. When the reinforcement slipped, the chemical cementation and the wrapping force provided by the fibers wrapped around the surface of the reinforcement, and the fibers at the other end inside the concrete matrix jointly provide a “rope” resistance for the reinforcement, which effectively delays the pullout speed of the reinforcement and finally makes the bond–slip curve slowly decline. (2) As shown in Figure 9 (a) SSC-GF_0.2_5D-R, (b) SSC-PPF_0.3_5D-R, and (c) SSC-GF_0.15_PPF_0.15_5-D-R, the pullout splitting and splitting failure only occur in the ascending segment, and the initial development of bonding stress is similar to that of the pullout failure, which requires no further elaboration With the continual increase in load, the bonding stress ascends until it reaches the ultimate stress state, and the bonding stress continued to increase until it reached the ultimate stress state. Here, the bonding stress was the highest and had a slight downward trend as the load increases. Then, the specimen cracked and the test ended. The reason is that when the pullout specimen was subjected to tensile stress, the micro-cracks first appeared inside the reinforcement and continued to develop, which accelerated the cracking rate due to the large elastic modulus of concrete. The appearance of the concrete specimen did not show obvious damage at this time, but as the cracks continued to develop, the specimen rapidly split into several pieces. Compared with the splitting failure of the SSC group in the control group without fiber doped that has no inflection point in the bond–slip curve, the specimen with fiber doped had improved the predictability of specimen failure; i.e., the bond–slip has an obvious inflection point.

In summary, any kind of fiber can delay the cracking failure time or change the failure mode of the specimen to a certain extent after the SSC is doped, so the probability of failure itself is predictable.

#### 4.2.2. Effect of Bond Length on Bond–Slip Curve

**Figure 10:**
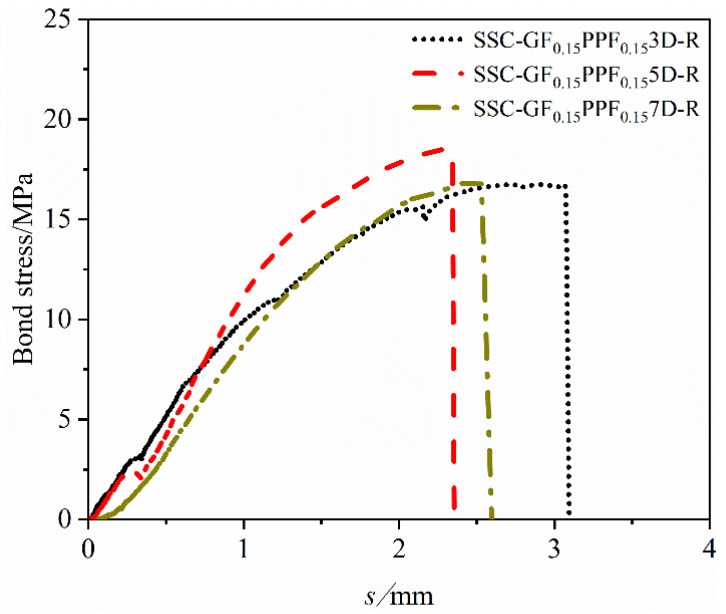
Effect of different bond lengths of BFRP bars on the bond–slip curve.

Figure 10 reflects the effect of varying bond lengths on the bond–slip curve of BFRP bars and SSC. This analysis adopts the method of controlling variables. The blending ratio is controlled to 1:1, and the GF and PPF content of the threaded reinforcement are both 0.15%. It can be considered that when the bond lengths are set to 3D, 5D, and 7D, respectively, the bond–slip curve being studied is not affected by other factors.

It can be seen from the figure that as the bond length increases, the corresponding peak bonding stress first increases and then decreases. This shows that the bond length of 5D can best reflect the actual bonding performance between BFRP bars and SSC, and that an excessive bond length will increase the stress transmission path of the reinforcement and the probability of nonlinear stress transmission, and it is more likely to cause stress concentration and uneven distribution, thereby reducing the bonding performance between the reinforcement and concrete.

#### 4.2.3. Effect of Surface Characteristics on Bond–Slip Curve

**Figure 11:**
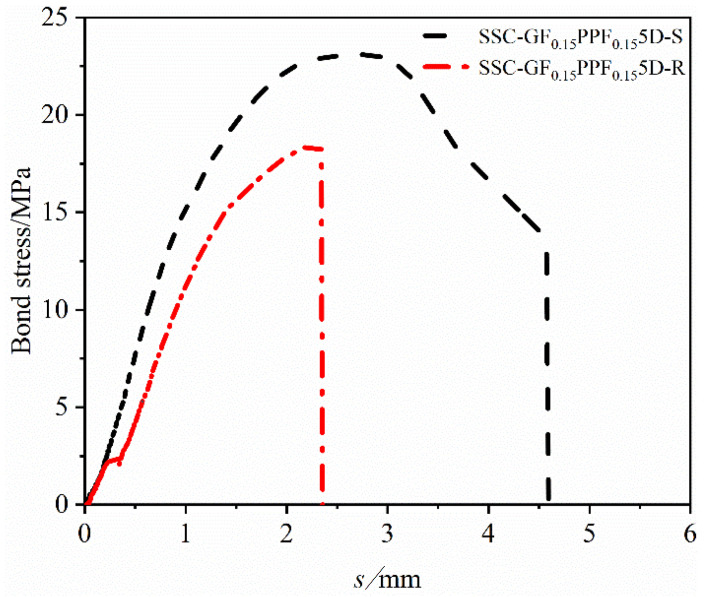
Effect of different surface characteristics of BFRP bars on bond–slip curve.

Figure 11 is a bond–slip curve drawn based on the effect of different BFRP bar surface characteristics on SSC bonding performance. By controlling the blending ratio to 1:1 and keeping the threaded reinforcement unchanged, it can be considered that the effect of the set surface characteristics of the reinforcement surface sandblasting and thread winding on the bond–slip curve is not affected by other factors. It can be seen from the figure that when other influencing factors remain unchanged, the peak bond stress between BFRP bars and SSC of sandblasted reinforcement was greater than that of threaded reinforcement, and the failure form of the sandblasted reinforcement test group was the reinforcement pullout failure with good ductility effect, while that of the threaded reinforcement test group occurred the concrete splitting failure with relatively greater brittleness. We found the following reasons. (1) The surface roughness of the surface sandblasted reinforcement is greater, which increases the friction between the reinforcement and concrete, thereby increasing the peak bond stress; on the other hand, during the slip stage, the fine sand on the surface partially falls off, which reduces the friction between the reinforcement and the concrete, causing a “ball effect”. This makes the reinforcement easier to pull out during the test, prolongs the failure time of the specimen, and has better ductility. (2) In the test, after the chemical bonding force between the reinforcement and concrete was completely lost, the actual stress process of the threaded reinforcement only relied on the mechanical snap-in force between the threads and concrete as well as the friction between the reinforcement and concrete [45,46]. Due to the fact that the friction force was lower than that of the sandblasting group, the indirect test peak bond force was lower than that of the sandblasting group. When the threaded ribs extruded on the surface of the threaded reinforcement were subjected to stress, they usually sheared the concrete embedded in the bonding transition zone. The cracks that occurred during this process developed rapidly, resulting in splitting failure and brittleness, which made it relatively difficult to predict the safety in engineering practice.

## 5. SEM Microscopic Mechanism Analysis

**Figure 12:**
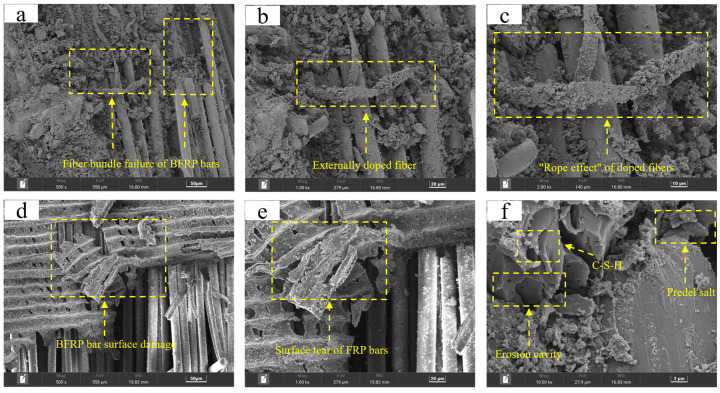
SEM microscopic image. (**a**–**c**) The rope effect of fibers on fiber tendon clusters. (**d**–**e**) The surface damage characteristics of BFRP bars. (**f**) The internal generation of concrete and destruction of pores.

Figure 12 is an SEM image of the transition zone at the interface between reinforcement and concrete. From the relationship between microstructure and material, we can more intuitively observe the reasons for the failure mode conversion of BFRP bars and SSC during the pullout test, and we can also observe how the doped fibers improved the bonding performance between reinforcement and concrete. It can be observed from Figure 12a–c that during the pullout test of BFRP bars, even when the outer fibers of the reinforcement are broken or cracked, the inner fiber bundles are still unaffected. This is because the doped fibers form the chaotic entanglements on the surface of the reinforcement and the transition zone of the concrete, so even if part of the fiber bundle of the fiber reinforcement is broken, the externally chopped continuous fibers can still play the role of “stirrups”, which ensures that the internal fiber bundle of the fiber reinforcement will not be damaged or destroyed; on the other hand, when the externally chopped continuous fibers are subjected to tensile stress, the fibers wrapped around the surface of the fiber reinforcement and existed in the interface transition zone between concrete and reinforcement act as the “rope resistance” to the pullout specimen, which plays a certain role in changing the failure mode of the pullout specimen from brittle failure to ductile failure.

It can be seen from Figure 12d,e that the resin layer on the outer surface of the sandblasted BFRP bars shows a tearing phenomenon, which explains that the sandblasted BFRP bars and SSC suffered more pullout failure of the reinforcement in the pullout test. It can further explain that when the bonding stress is applied to the interface transition area between the fiber reinforcement and concrete, the bonding stress of the sandblasting group is mainly provided by the friction force between reinforcement and concrete rather than the mechanical biting force similar to that of threaded reinforcement. Therefore, the friction between the reinforcement and the concrete will increase as the bonding stress continues to increase until the surface resin layer is torn apart and the reinforcement gradually slips out. As time goes by, the pullout failure with good ductility will occur, causing the reinforcement to be pulled out of the concrete, which is accompanied by a “hissing” sound. On the contrary, in the threaded bar test group, the thread mainly provided mechanical snap-in force to increase the bonding stress during the test. But usually, as the test load continues to increase, the mechanical snap-in force of the concrete in the transition zone between the thread and the interface is not enough to provide strong and effective bonding stress, and then the interlaminar shear failure of the concrete occurs, and cracks develop rapidly. The final failure mode of the specimen was a sudden brittle failure, which is accompanied by the crisp sound of concrete cracking.

It can be seen from Figure 12f that there are many salt crystals and “Fredel salt” inside the SSC matrix to fill the concrete matrix [47,48]. The dense C-S-H hydration products provide the dense internal structure of the concrete matrix. However, due to the existence of harmful substances such as chlorine salts and sulfates in seawater, the dense C-S-H hydration products have been eroded, resulting in more porous structures, making the matrix loose and porous, and causing the sudden brittle failure of most of the pullout specimens in this test. This is consistent with the research results of Professor Xiao et al. [49]. The doped fiber can increase the toughness of concrete to a certain extent, but the appropriate fiber content and type are the key.

## 6. Bond–Slip Constitutive Relationship Model between BFRP Bars and SSC

### 6.1. Model of Bond–Slip Constitutive Relationship

The bond–slip constitutive relationship model is a basic study of the bonding performance between BFRP bars and SSC. Many scholars have modified and verified the bond–slip constitutive relationship model between steel reinforcement and normal-strength concrete repeatedly and obtained the bond–slip constitutive relationship model between GFRP reinforcement and concrete with high correlation and reliability. Here, we include the main foreign research results. Malvar [50] first proposed the bond–slip constitutive relationship model between GFRP reinforcement and concrete in 1994 based on the bond–slip curve characteristics displayed during the specimen test; in 1995, Cosenza et al. combined numerous experimental bond–slip curves, mainly on the ascending segment of the curve, and finally obtained the CMR [51] model. In 1997, the fitting of the horizontal segment of the bond–slip curve in the BPE constitutive relationship model [52] was eliminated, making the bond–slip curve fitted by the entire model more in line with the development trend of the bond–slip curve in the actual situation, thus obtaining the improved BPE [53]. Domestic scholars Gao et al. [54] believe that among many models, the ascending and descending segments and the peak point of the bond–slip curve in the constitutive relation model are not continuous smooth curves. Therefore, a constitutive relation model of continuous curves was proposed based on the advantages of the models obtained by various scholars.

Substituting the bond–slip data into the above model for fitting verification shows that there is a certain degree of correlation with the experimental data. However, the degree of fitting is not particularly high, and the disparity in the data is relatively substantial. By recombining the ascending and descending segments of various models, the combination of the ascending segment of the Malvar model and the descending segment of the improved BPE model was achieved, which shows that the fitting degree of the bond–slip curve data of this test is relatively high. Therefore, in the case of a bond–slip curve featuring solely an ascending segment, the ascending segment of the Malvar model is utilized. Meanwhile, for a two-stage bond–slip curve comprising both an ascending and a descending segment, a composite of the two models is applied. The specific expression for the constitutive relationship model is delineated in Equations (2) and (3).

(2) $$\;\text{Ascending}\;\text{segment}:\;s\leq s_{1}\;\;\frac{\tau }{\tau_{1}}=\frac{F(\frac{s}{s_{1}})+(G-1){\left(\frac{s}{s_{1}}\right)}^{2}}{1+(F-2)(\frac{s}{s_{1}})+G{\left(\frac{s}{s_{1}}\right)}^{2}}$$

(3) $$\text{Descending}\;\text{segment}:\;s_{1}\leq s\;\;\frac{\tau }{\tau_{1}}=1-P(\frac{s_{1}}{s}-1)$$

In which:

*τ*_1_—peak bonding stress (MPa);

*s*_1_—the slip amount corresponding to the peak bonding stress (mm);

*F*,*G*,*P*—empirical parameters determined by the test.

### 6.2. Model Verification

Substituting the bond–slip data obtained from the test into Equations (2) and (3) according to categories, and using Origin software (2018) to perform the data fitting, the resultant fitting diagram and pertinent empirical parameter tables were obtained.

**Figure 13:**
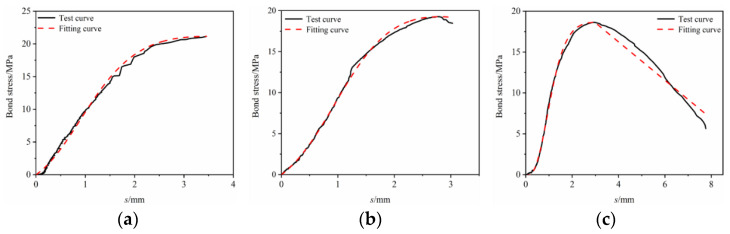
Constitutive relationship model fitting under the effect of GF. (**a**) SSC-GF_0.1_-R-5D; (**b**) SSC-GF_0.2_-R-5D; (**c**) SSC-GF_0.3_-R-5D.

**Figure 14:**
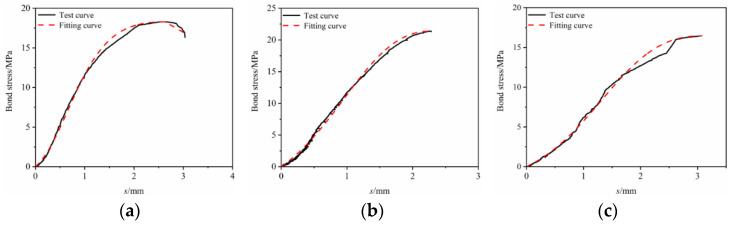
Constitutive relationship model fitting under the effect of PPF. (**a**) SSC-PPF_0.1_-R-5D; (**b**) SSC-PPF_0.2_-R-5D; (**c**) SSC-PPF_0.3_-R-5D.

**Figure 15:**
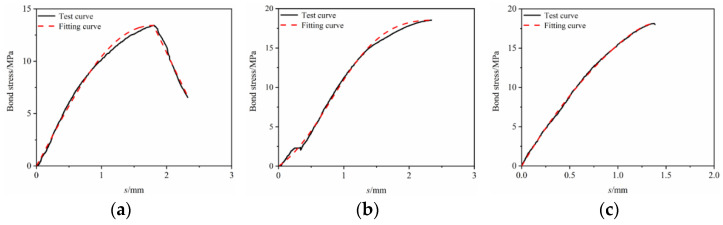
Constitutive relationship model fitting under the effect of hybrid fibers. (**a**) SSC-GF_0.1_ PPF_0.2_-R-5D; (**b**) SSC-GF_0.15_ PPF_0.15_-R-5D; (**c**) SSC-GF_0.2_ PPF_0.1_-R-5D.

**Figure 16:**
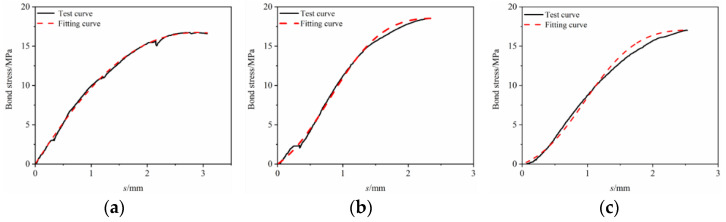
Constitutive relationship model fitting under the effect of different bond lengths. (**a**) SSC-GF_0.15_ PPF_0.15_-R-3D; (**b**) SSC-GF_0.15_ PPF_0.15_-R-5D; (**c**) SSC-GF_0.15_ PPF_0.15_-R-7D.

**Figure 17:**
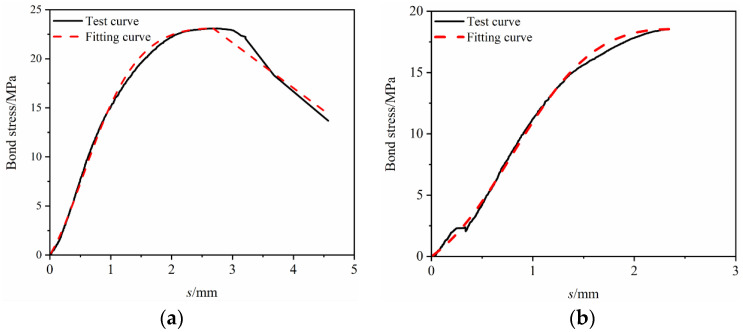
Constitutive relationship model fitting under the effect of surface features. (**a**) SSC-GF_0._**_1_**_5_ PPF_0.15_-S-5D; (**b**) SSC-GF_0.15_ PPF_0.15_-R-5D.

The constitutive relationship model was fitted to the bond–slip test data. It can be seen from Figure 13, Figure 14, Figure 15, Figure 16 and Figure 17 that the fit curve has a high coincidence degree with the bond–slip curve obtained by the test, and the correlation coefficients R^2^ obtained in Table 8 are all greater than 0.98. This indicates that the model recombined according to the ascending segment of the Malvar model and the descending segment of the improved BPE model has a high degree of fitting for this test, so it can provide a reference for the study of the constitutive relationship between BFRP bars and seawater sand fiber-reinforced concrete.

## 7. Conclusions

The sustainable development of the construction industry is the future direction of development. The investigation of seawater sand concrete is bound to emerge as a focal point of research interest, and the bonding performance of steel and concrete is a critical indicator of the regular operation of reinforced concrete structures. Therefore, we have performed the following work. The test mainly studied the effect on the bonding properties between BFRP bars and SSC when the volume content of GF and PPF fibers was 0.1%, 0.2%, and 0.3%, the hybrid ratio of blending fibers was 1:2, 1:1, and 2:1, the bond length of BFRP bars was 3D, 5D, and 7D, and the surface features of the reinforcement are encompassed sandblasting and threads. The following conclusions are obtained by specific research and analysis:

(1) Compared with the control group without fiber, the bond strength of the samples doped with fiber is generally better, but the effect of different kinds of fiber on the bond strength is different. Compared with SSC in the control group, the most obvious improvement effect of mono-doped GF and PPF on bond strength was 0.1% and 0.2% content, respectively, which is increased by 40.40% and 41.65%. For blended GF and PPF, the bond strength is increased by 22.51% when the blending ratio is 1:1.
(2) Compared with the control group SSC without fiber doped, it can be seen from the bond–slip curve that the slip time is relatively prolonged after the fiber is doped, leading to enhanced prognostication of failure. However, it is noteworthy that a higher fiber content does not necessarily yield superior outcomes. Excessive fiber content may diminish the bonding characteristic of the specimen due to uneven blending, inadequate compaction, and suboptimal dispersion.
(3) The highest bonding strength is shown when the bond length is 5D. As the bond length increases from 3D and 5D to 7D, the bond strength first increases and then decreases, and the bonding strength of the sandblasted fiber bars after adding fiber was higher than that of the thread group..
(4) It can be seen from the bond–slip curve that most specimens have splitting failure, and a few have pullout failure. The reason is that SSC has a high elastic modulus, and the internal voids or pores are filled with “Fredel salt” generated by the residual salt crystals of seawater sand and the hydration products of chlorine salt, sulfate, and cement. Even if the fiber is doped, due to its low viscosity and high brittleness, the gripping force of the matrix on the fiber is reduced, and the “tension–compression stress” of the specimen on the steel plate counter-force cage is prone to brittle failure.
(5) The combination of the ascending segment of the Malvar model and the descending segment of the improved BPE model is finally obtained through the data fitting of many classical bond–slip constitutive relationship models. The bond–slip curve data of this test have a high degree of fitting, and the correlation coefficients are all greater than 0.98, which has a high correlation and can provide certain theoretical guidance for the engineering applications of BFRP bars and SSC.
(6) In the future, it will be possible to test the effect of compression strength, freeze–thaw cycles, and other factors on the bonding properties of reinforcement materials to seawater sand concrete.

## Figures and Tables

**Table 1:** Performance indexes of BFRP reinforcements.

Reinforcement Type	Length/cm	Elastic Modulus/GPa	Yield Strength/MPa	Diameter/mm	Elongation/%	Density/(g/cm^3^)
BFRP	50	51.6	1266	12	2.75	1.96

**Table 2:** Chemical composition of seawater.

Ion Type	Ca^2+^	Mg^2+^	Na^+^	K^+^	${{SO}_{4}}^{2-}$	Cl^−^	${{HCO}_{3}}^{-}$
Ion concentration (mg/L^−1^)	264	963	7848	227	2014	14541	335

**Table 3:** Chemical composition of cement.

Item	Chemical Composition Content/%					
	Al_2_O_3_	CaO	K_2_O	MgO	SiO_2_	TiO_2_
Cement	66.30	19.60	6.50	3.50	2.50	0.70

**Table 4:** Basic mechanical properties of cement (MPa).

3 d Compressive Strength	3 d Flexural Strength	28 d Compressive Strength	28 d Flexural Strength
5~20	1615	2655	8.80

**Table 5:** Physicochemical indexes of natural aggregate.

Particle Size of Aggregate/mm	Volume Density (kg/m^3^)	Apparent Density (kg/m^3^)	Crushing Index/%	Mud Content/%	Needle Flake Content/%
5~20	1615	2655	8.80	0.32	3.65

**Table 6:** Fiber performance index.

Fiber Type	Tensile Strength/MPa	Elastic Modulus/GPa	Length/mm	Density/(g/cm^3^)
GF	1900~2500	75~100	12	2.68
PPF	400~550	≤4	12	0.91

**Table 8:** Fitting results of relevant parameters of the constitutive relationship model.

Group	No.	F	G	P	R^2^
1	SSC-R-5D	0.659	0.580	/	0.982
2	SSC-GF_0.1_-R-5D	0.865	3.013	/	0.990
3	SSC-GF_0.2_-R-5D	0.730	1.950	/	0.999
4	SSC-GF_0.3_-R-5D	−0.250	4.912	0.375	0.997
5	SSC-PPF_0.1_-R-5D	0.802	3.517	0.511	0.997
6	SSC-PPF_0.2_-R-5D	0.788	1.034	/	0.985
7	SSC-PPF_0.3_-R-5D	0.632	1.325	/	0.981
8	SSC-GF_0.1_ PPF_0.2_-R-5D	1.550	0.456	1.762	0.996
9	SSC-GF_0.15_ PPF_0.15_-R-3D	1.910	0.404	/	0.998
10	SSC-GF_0.15_ PPF_0.15_-R-5D	0.735	1.897	/	0.997
11	SSC-GF_0.15_ PPF_0.15_-R-7D	0.480	2.074	/	0.993
12	SSC-GF_0.15_ PPF_0.15_-S-5D	1.202	3.360	0.536	0.998
13	SSC-GF_0.2_ PPF_0.1_-R-5D	1.588	−0.366	/	0.999

## Data Availability

Data are contained within the article.
